# Acknowledgement to Reviewers of *Membranes* in 2019

**DOI:** 10.3390/membranes10010018

**Published:** 2020-01-19

**Authors:** Membranes Editorial Office

**Affiliations:** MDPI AG, St. Alban-Anlage 66, 4052 Basel, Switzerland

The editorial team greatly appreciates the reviewers who have dedicated their considerable time and expertise to the journal’s rigorous editorial process over the past 12 months, regardless of whether the papers are finally published or not. In 2019, a total of 173 papers were published in the journal, with a median time to first decision of 14.00 days and a median time from submission to publication of 37.00 days. The editors would like to express their sincere gratitude to the following reviewers for their generous contribution in 2019:
Abdala, AhmedLiu TianyuAbejón, RicardoLo, Huang-MuAcquah, SteveLongo, Maria A.Ajao, OlumoyeLoulergue, PatrickAlbo, JonathanLow, NicholasAltaee, AliLucena, RafaelAn, QuanfuLyu, HailongAnsaloni, LucaMaas, MichaelArkas, MichaelMadden, DavidArunkumar, AbhiramMagnacca, GiulianaAyyavoo, JayalakshmiMagnico, PierreBalasingam, Suresh KannanMaior, IoanaBazhenov, Stepan D.Maletskyi, ZakharBazzarelli, FabioMansouri, AbrahamBell, Carl-MartinMareev, SemyonBengtson, GiselaMartí-Calatayud, Manuel CésarBermeshev, MaximMartin, Stephen M.Bi, LeiMasoudi Soltani, SalmanBogdanowicz, Krzysztof ArturMather, GlennBoo, ChanheeMazzei, RosalindaBouwman, WimMedrano Jimenez, Jose A.Breite, DanielMelnikov, StanislavBrinkmann, TorstenMenendez, MiguelBrunklaus, GuntherMeng, LieBrutti, SergioMerino-Garcia, IvanBulejko, PavelMilčius, DariusCamões, Maria FilomenaMiller, Daniel N.Cannilla, CatiaMinamiki, TsukuruCao, BingMinelli, MatteoCao, BoMonteiro, BernardoCarreon, MoisesMozia, SylwiaCasado-Coterillo, ClaraMurmura, Maria AnnaCasares, JuanMurtomäki, LasseCassano, AlfredoNagy, EndreCastro-Muñoz, RobertoNakielski, PawelChalker, JustinNardi, MonicaChang, HsuanNiakolas, Dimitrios K.Checchetto, RiccardoNicoletta, Fiore PasqualeChen, JinhuaNikbakht Fini, MahdiCheng, GangNikolelis, Dimitrios P.Cheng, LishengNikonenko, VictorChetwynd, AndrewNoor, NuruzzamanCho, KangwooNowaczyk, JacekChun, YoungpilNtougias, SpyridonChung, Neal Tai-ShungOkamoto, YoshiyukiChung, Sheng-HengOsman, AhmedCoronas Ceresuela, JoaquínPacheco Tanaka, David AlfredoCortina, Jose LuisPacheco, FedericoCosta, Carlos MiguelPadmanabhan, VenkatCowan, Matthew GreigPalma, LucaCran, Marlene J.Palmer, ChristopherCui, ZhaoliangPanglisch, StefanD’Haese, ArnoutPaolone, AnnalisaDa’na, EnshirahPapadakis, RaffaelloDai, ZhongdePapangelakis, VladimirosDammak, LasaadPark, Chul-hoDe Schepper, WimPark, Jin-SooDeimede, ValadoulaPatinvoh, ReginaDeng, HuiningPatsios, SotirisDeng, LiyuanPeng, XinshengDesfougères, YannPereira, Cláudia C. L.Dhamdhere, AjitPetrella, AndreaDi Profio, GianlucaPulyalina, AlexandraDing, YifuRajca, MariolaDobrzynski, PiotrRaju, KatiDong, YingchaoRehder, DieterDutournié, PatrickRincón, Marina E.Dutta, Ravi ChandraRodríguez-Roda, IgnasiEliseeva, TatianaRogozhnikov, Denis A.Escorihuela, JorgeRonen, AvnerEskelin, KatriRoubík, HynekEsrafilzadeh, DornaRowlett, JarrettEstay, HumbertoRoy, SagarFeng, HongboRuiz De Larramendi, IdoiaFernandes, Mariana Sofia PeixotoRuiz-García, AlejandroFievet, PatrickRupprich, MarcoFontananova, EnricaSaakes, MichelFontàs, ClaudiaSaeki, DaisukeFranzese, GiancarloSakaguchi, ToshikazuFuoco, AlessioSalamanca, Constain H.Gaglione, RosaSamhaber, W. M.Gallucci, FaustoSantoro, SergioGao, PeiyuanSanyal, OishiGarcia Ivars, JorgeSarafraz, Mohammad MohsenGarcía-Moncada, NuriaSavaniu, CristianGeng, LinxiaoSawamura, Ken-ichiGeorgopanos, ProkopiosScalese, SilviaGhatak, KamalikaSchweiss, RuedigerGhoshal, ShraboniSelyanchyn, RomanGierszewska, MagdalenaSemsarilar, MonaGlüsen, AndreasSentana, IreneGoh, KunliShamsaei, EzzatollahGomez-Coma, LuciaShao, LuGroves, MatthewSharma, SurbhiGryta, MarekShen, JiangnanGueorguiev, GueorguiShevate, RahulGupta, MohitShishatskiy, SergeyGupta, SanjuShon, HokyongHajizadeh, SolmazSiebenhofer, MatthäusHarmand, JérômeSilva, RicardoHarvianto, GregoriusSinha Ray, SaikatHaugen, Astri BjørnetunSioutopoulos, DimitriosHe, GuangweiSkorik, Yury A.He, ShaojianSlouka, ZdenekHe, WeilueSong, XiaoxiaoHeath, GeorgeSousa, AngelaHeimburg, ThomasStoller, MarcoHelmi, ArashSt-Pierre, JeanHernández, AntonioStuckey, David C.Hidalgo, Asuncion MariaSzekely, GyorgyHiga, MitsuruTadyszak, KrzysztofHirota, YuichiroTanaka, ShunsukeHolze, RudolfTańczyk, MarekHomaeigohar, ShahinThibault, JulesHong, LiangTiraferri, AlbertoHong, TaoTong, XinHou, JingweiTrukhanov, Sergei ValentinovichHuang, XiaowuTsapatsis, MichaelIbáñez Mendizábal, RaquelTung, Kuo-LunIkeda, TaichiTuzimski, TomaszIliev, BoyanTylkowski, BartoszItta, ArunUetani, KojiroIulianelli, AdolfoUzdenova, AminatJacob, PaulValencia, SusanaJacob, PaulVan Goethem, CédricJansen, JohannesVan Sint Annaland, MartinJay-Gerin, Jean-PaulVanangamudi, AnbharasiJeerapan, ItthiponVandezande, PieterJeong, SanghyunVenturi, DavideJi, GuozhaoVincent-Vela, Maria-CintaJiang, ChenxiaoVolkov, AlexeyJiang, YingbingVolkov, VladimirJin, JianyongVorotyntsev, Ilya V.Jones, DeborahVrouwenvelder, JohannesJun, Young-SiWachowski, SebastianJung, Sokhee PhilemonWang, DavidKallitsis, JoannisWang, ZhaoxuKang, Dun-YenWang, ZhengbaoKang, Sang WookWang, ZhiweiKarahan, H. EnisWieczorek, PiotrKaranikola, VasilikiWilson, Aaron DKarp, Eric M.Woldemariam, DanielKe, XieWood, Jeffery A.Kerman, KaganWu, BingKern, WolfgangWu, Gwo-MeiKertèsz, SzabolcsWu, ZhentaoKhorshidi, BehnamWycisk, RyszardKim, Ae RhanXu, ZhiweiKim, DukjoonXue, JinkaiKim, HankiXue, XingjianKim, Han-SeungYang, HsiharngKim, HyunsungYang, ShaoweiKim, Seok-JhinYoo, ChangkyooKim, Sung-KooYoo, Chung-YulKim, Tae-hwanYoo, Dong JinKochetova, Nadezhda AlexandrovnaYoshihiko, SanoKononova, Svetlana V.Yu, HaoranKoter, StanislawYu, JaecheulKritikos, GeorgiosYu, LiangKruczek, BoguslawZarca, GabrielKujawa, JoannaZdenek, SloukaKujawski, WojciechZhang, HanKulesha, OlgaZhang, LuhongKvasha, AndriyZhang, XiangLade, Harshad S.Zhang, XiaoyuanLaoui, TaharZhang, XinLaskowski, ŁukaszZhang, XuyangLasseuguette, ElsaZhang, ZhienLau, Woei JyeZhang, ZhongqiangLee, Chia-HungZhao, LianfengLee, Dai-SooZhao, WeifengLee, Jong SukZheng, QingbinLee, Kew-HoZhou, MingLee, Pyung SooZhou, XingpingLi, HeZhou, ZhipingLi, JianxinZhu, GuanghuiLi, JunshengZhu, LingxiangLiang, ShuangZhu, MeihuaLillepärg, JelenaZhu, ShanLipnizki, FrankZouboulis, AnastasiosLipscomb, GlennZuo, JianLiu, Liang


